# Is Arsenic “Lactation Intolerant”?: Study Indicates Low Excretion in Breast Milk

**DOI:** 10.1289/ehp.116-a306b

**Published:** 2008-07

**Authors:** Tanya Tillett

Arsenic is known to readily cross the placenta, but few data exist on postnatal exposure to arsenic in breast milk. Results of a study conducted in Bangladesh now suggest that infants who are exclusively breastfed are protected against arsenic, despite high maternal exposures **[*EHP* 116:963–969; Fängström et al.]**.

Numerous studies have linked arsenic exposure in adults to various diseases, including cancer, cardiovascular disease, and diabetes mellitus. Exposure in school-age children has been associated with neurodevelopmental disorders. During fetal development, the brain is particularly vulnerable to arsenic exposure, as it readily crosses the placenta, possibly altering fetal programming and leading to a higher risk of susceptibility to disease later in life.

The subjects in the current study included 98 mothers and their 3-month-old infants who participated in the Maternal and Infant Nutrition Interventions of Matlab in Bangladesh, one of the most severely affected countries in terms of high prevalence of extremely elevated levels of arsenic in drinking water supplies. The investigators evaluated nutritional status and arsenic exposure as reflected by arsenic metabolites in infant urine and maternal blood, urine, and saliva samples. They also analyzed breast milk samples at 2 months postpartum for arsenic. Questionnaires completed by the mothers provided data on infant feeding practices.

The median sum of arsenic metabolites in infant urine was 1.2 μg/L, with significantly lower concentrations in infants who were exclusively breastfed compared with those who received some solid food. Arsenic concentrations in breast milk were low (median 1.0 μg/kg) and mostly in the form of trivalent inorganic arsenic. The researchers observed a significant association between arsenic in infant urine and breast milk, but noted that some mothers with low breast milk arsenic had infants with high urine concentrations, possibly because the infants had been given water to drink. Median maternal blood and urine concentrations were high (5.7 and 67 μg/L, respectively), whereas median maternal saliva concentrations were low (1.3 μg/L). Among infants who were exclusively breastfed, urine levels did not exceed 19 μg/L inorganic arsenic and its metabolites, whereas infants who received infant formula prepared with local drinking water in addition to some breast milk had urine levels up to 1,100 μg/L.

The authors demonstrate for the first time that arsenic in human breast milk is mostly the inorganic arsenite form. Although there was a significant relationship between arsenic concentrations in milk and in maternal blood, arsenic concentrations in breast milk were relatively low despite the mothers’ high exposures. The findings suggest that breastfeeding exclusively can protect infants from arsenic exposure during this critical development period, but the authors note that researchers have yet to determine the extent to which breastfeeding decreases the health risks associated with prenatal arsenic exposure.

